# Supplementary material for: High-fidelity spin qubit operation and algorithmic initialization above 1 K
Source: Nature. 2024 Mar 27;627(8005):772–7. doi: 10.1038/s41586-024-07160-2 (PMC10972758; doi:10.1038/s41586-024-07160-2)
Supplement: Supplementary file 1 — Supplementary Information [file 41586_2024_7160_MOESM1_ESM.pdf]

nature portfolio

https://doi.org/10.1038/s41586-024-07160-2

Supplementary information

High-fidelity spin qubit operation and algorithmic initialization above 1 K

In the format provided by the
authors and unedited

High-fidelity spin qubit operation and algorithmic initialisation above 1 K

SUPPLEMENTARY INFORMATION

Device	Valley excitation	Orbital excitation
This work	-	2.5 meV
Ref. [1] (Device F)	0.2–0.3 meV	-
Ref. [2]	0.2–0.8 meV	~ 2 meV
Ref. [3]	~ 0.5 meV	2.9 meV
Ref. [4] (Device A)	-	2.6 meV

Supplementary Table I. **Excitation energies.** Valley and orbital excitation energies extracted from four similar SiMOS devices [1–4].

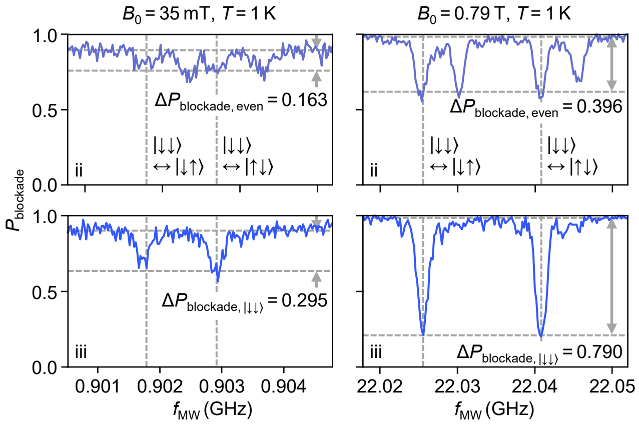

Supplementary Fig. 1 | Extraction of the algorithmic $|\downarrow\downarrow\rangle$ initialisation fidelity from the ESR spectra with exchange on. a, When exchange is on, the ESR transitions provide information about the two-qubit state composition, as labelled in Fig. 2 a. The first transition and the third transition from the left arise from the state transitions $|\downarrow\downarrow\rangle \leftrightarrow |\downarrow\uparrow\rangle$ and $|\downarrow\downarrow\rangle \leftrightarrow |\uparrow\downarrow\rangle$, and signify a $|\downarrow\downarrow\rangle$ state in the case of even-parity initialisation. With ideal spin inversion and readout, the amplitude of these two transitions yields an estimate of the fidelity of $|\downarrow\downarrow\rangle$ initialisation. The ESR spectra are measured by applying a microwave pulse at various frequencies and V_J. In order to fully invert the spins, the pulse duration is calibrated to be $t_{X_1(\pi)}$ at the single-qubit operation point. As the driving mechanism becomes different when exchange is on, this calibrated pulse does not fully invert the spins in these regimes. We first extract $\Delta P_{\mathrm{blockade,even}}$, the transition amplitude measured after Stage II of the algorithmic initialisation, which produces a mixed even-parity state, and $\Delta P_{\mathrm{initialisation},|\downarrow\downarrow\rangle}$, the transition amplitude measured after Stage III of the algorithmic initialisation, which produces a $|\downarrow\downarrow\rangle$ state. The initialisation fidelity is given by $F_{\mathrm{initialisation},|\downarrow\downarrow\rangle} = \Delta P_{\mathrm{initialisation},|\downarrow\downarrow\rangle}/(\Delta P_{\mathrm{blockade,even}}/0.5)$. We obtain $F_{\mathrm{initialisation},|\downarrow\downarrow\rangle} = 90.77\,\%$ and $F_{\mathrm{initialisation},|\downarrow\downarrow\rangle} = 99.56\,\%$ from the results at $B_0 = 35$ mT and $B_0 = 0.79$ T. See Supplementary Fig. 2 for the full ESR spectra.

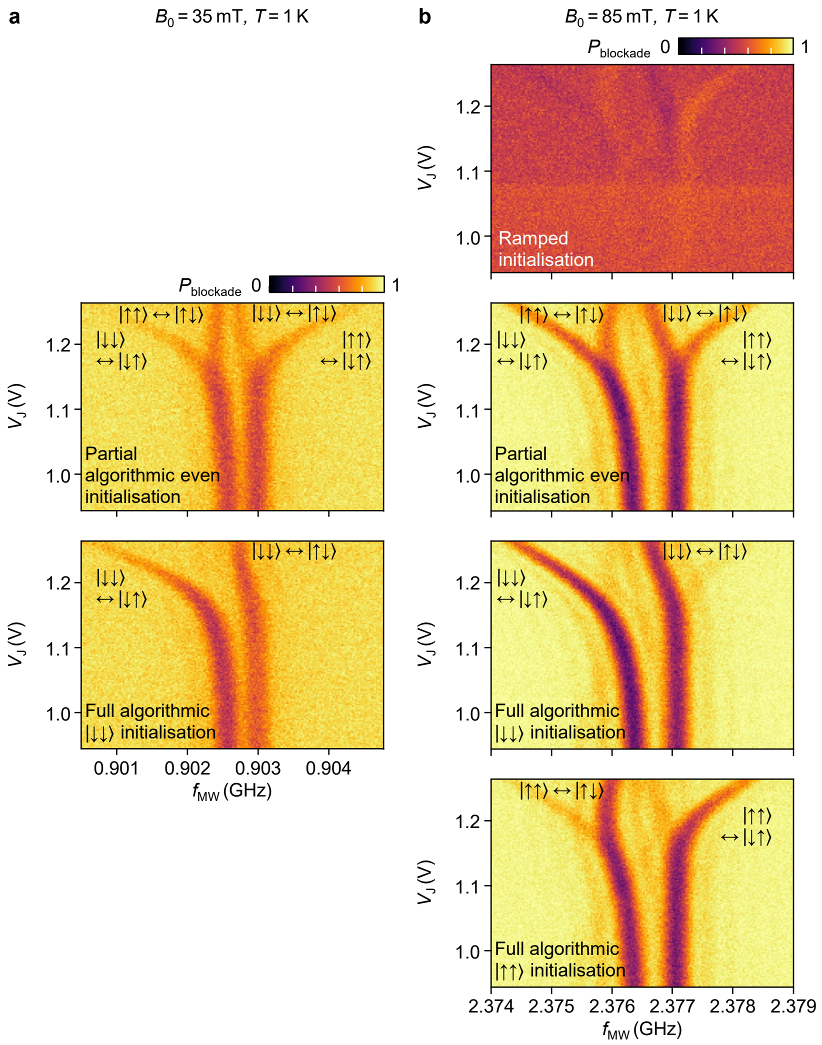

Supplementary Fig. 2 | ESR spectra as a function of V_J with different initialisation methods. **a**, $B_0 = 35\,\text{mT}$ and $T = 1\,\text{K}$, where the thermal energy is 20 times greater than the qubit energies. **b**, $B_0 = 85\,\text{mT}$ and $T = 1\,\text{K}$, where the thermal energy is 8 times greater than the qubit energies. **c**, $B_0 = 0.79\,\text{T}$ and $T = 1\,\text{K}$, where the thermal energy is near the qubit energies.

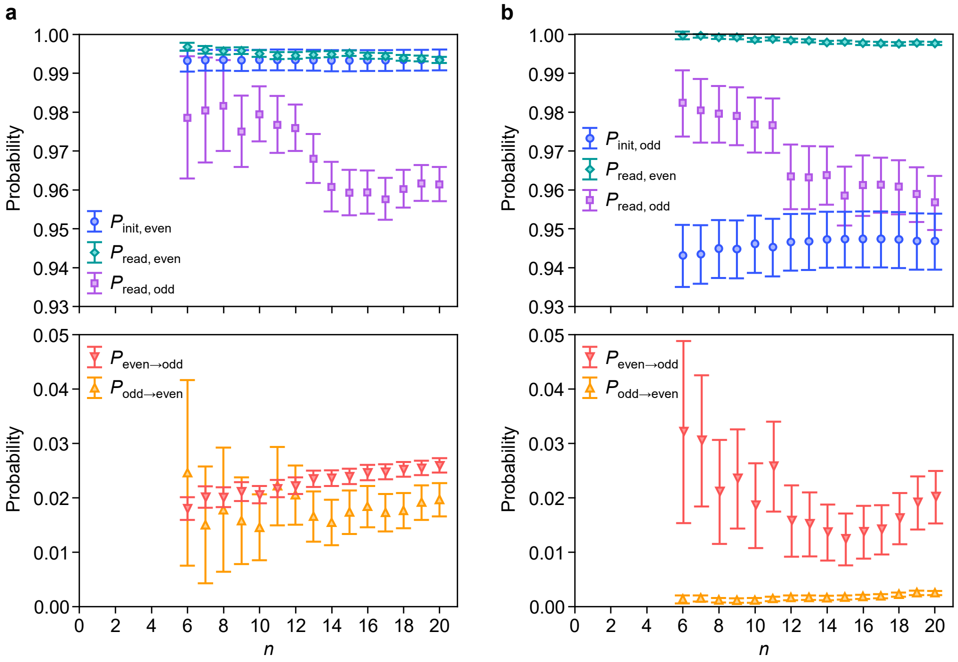

Supplementary Fig. 3 | Full results from SPAM analysis with machine learning. **a**, All SPAM probabilities inferred from repeated readout outcomes through machine learning, for algorithmic $|\downarrow\downarrow\rangle$ initialisation at $B_0 = 0.79\,\mathrm{T}$ and $T = 1\,\mathrm{K}$. **b**, All SPAM probabilities inferred from repeated readout outcomes through machine learning, for algorithmic $|\uparrow\downarrow\rangle$ initialisation at $B_0 = 0.79\,\mathrm{T}$ and $T = 1\,\mathrm{K}$. Error bars represent the $95\,\%$ confidence level.

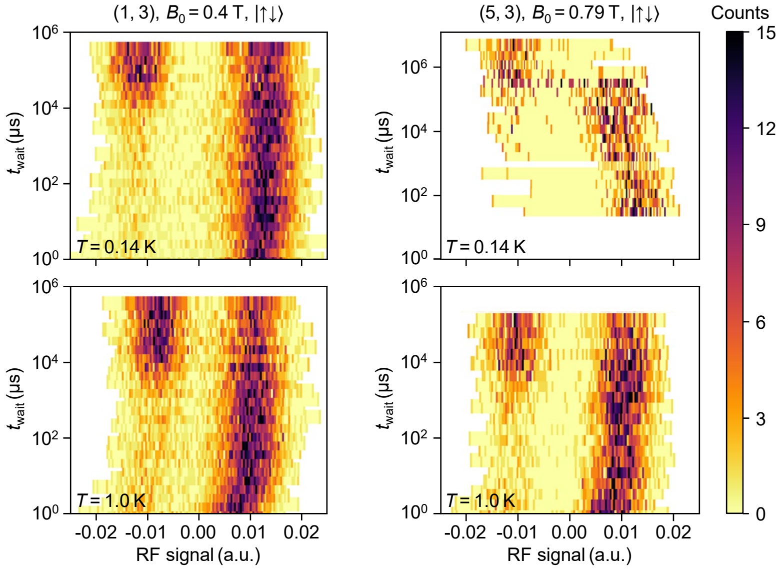

Supplementary Fig. 4 | Readout histograms as a function of time. Example readout histograms of long T_1 measurements as a function of time.

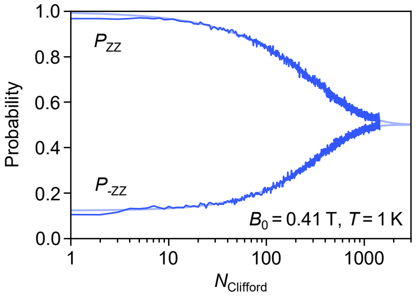

Supplementary Fig. 5 | +Z and −Z projections for a long single-qubit randomised benchmarking run. We note that spin relaxation, excitation, incorrect rotation or slow drifts in charge readout can obscure long sequence runs. Under our operating conditions at $T = 1\,$K, the longest randomised benchmarking sequences in our experiment reach an average of 1400 elementary gates, taking up to several hundreds of microseconds. This is well within the T_1 we measure. We measure the decay in the +ZZ (no operation before parity readout) projection and the −ZZ projection (π pulse on a single qubit before parity readout) after the recovery gate. With these projections, randomised benchmarking decays at about the same rate towards the same equilibrium of 0.5. The fitted Clifford fidelities from P_{Z}, $P_{-\mathrm{Z}}$ and $P_{\mathrm{Z}} - P_{-\mathrm{Z}}$ are $99.8484 \pm 0.0021\,\%$, $99.8547 \pm 0.0016\,\%$ and $99.8519 \pm 0.0013\,\%$. With this particular device configuration and the long physical gate sequences, the odd-parity readout fidelity is compromised. Error bars represent the 95 % confidence level.

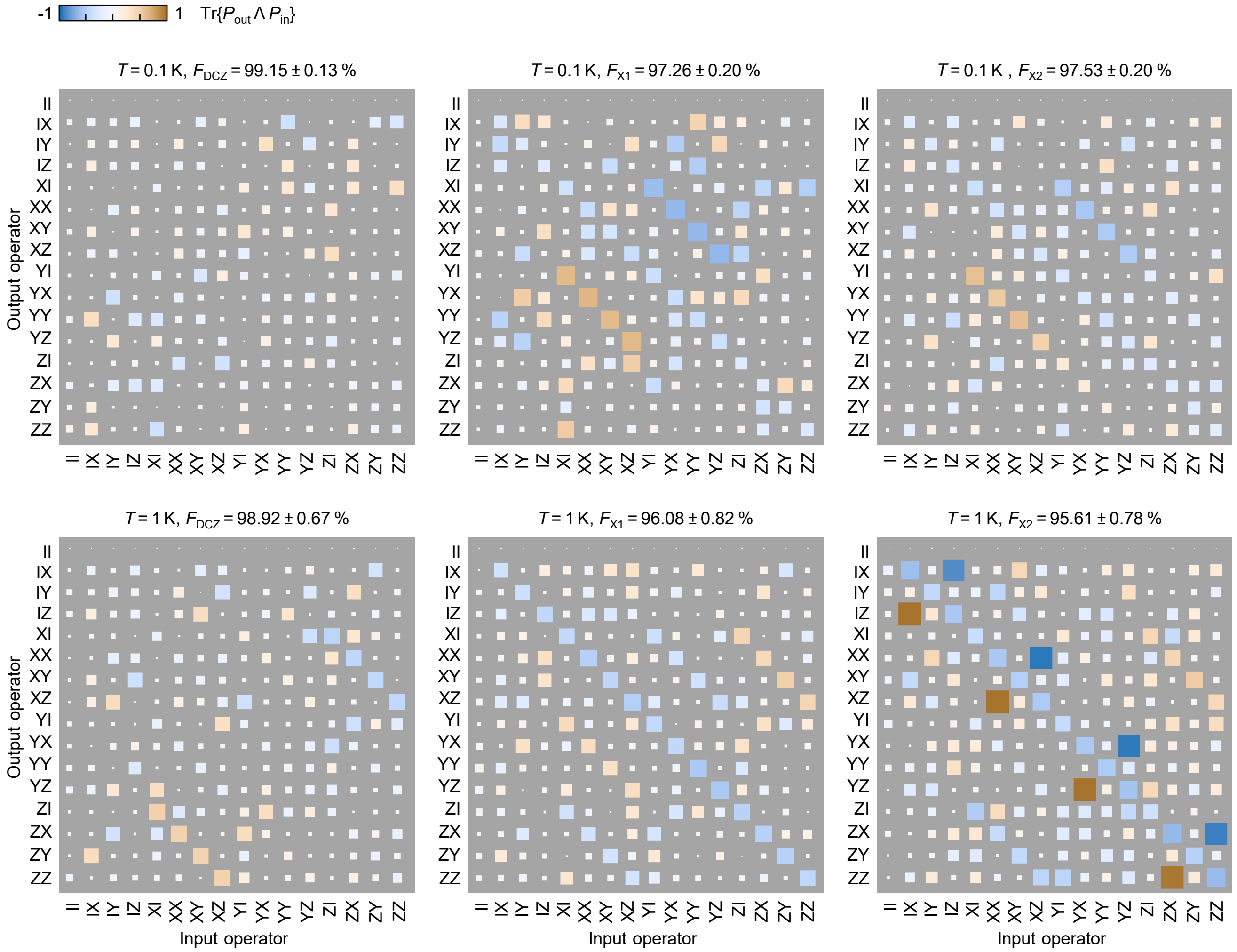

Supplementary Fig. 6 | FBT estimated noise residual PTMs. Projected error residual process matrices for the DCZ, $\mathrm{X}_1(\pi/2)$ and $\mathrm{X}_2(\pi/2)$ gates at $B_0 = 0.79\,$T, $T = 0.1\,$K and $1\,$K, extracted by FBT. We also show the consequent process fidelity for each gate. We note that when running two-qubit random sequences, a single-qubit gate on one qubit always leaves the other qubit idling, which leads to the low single-qubit process fidelities shown here. Error bars represent the 95 % confidence level.

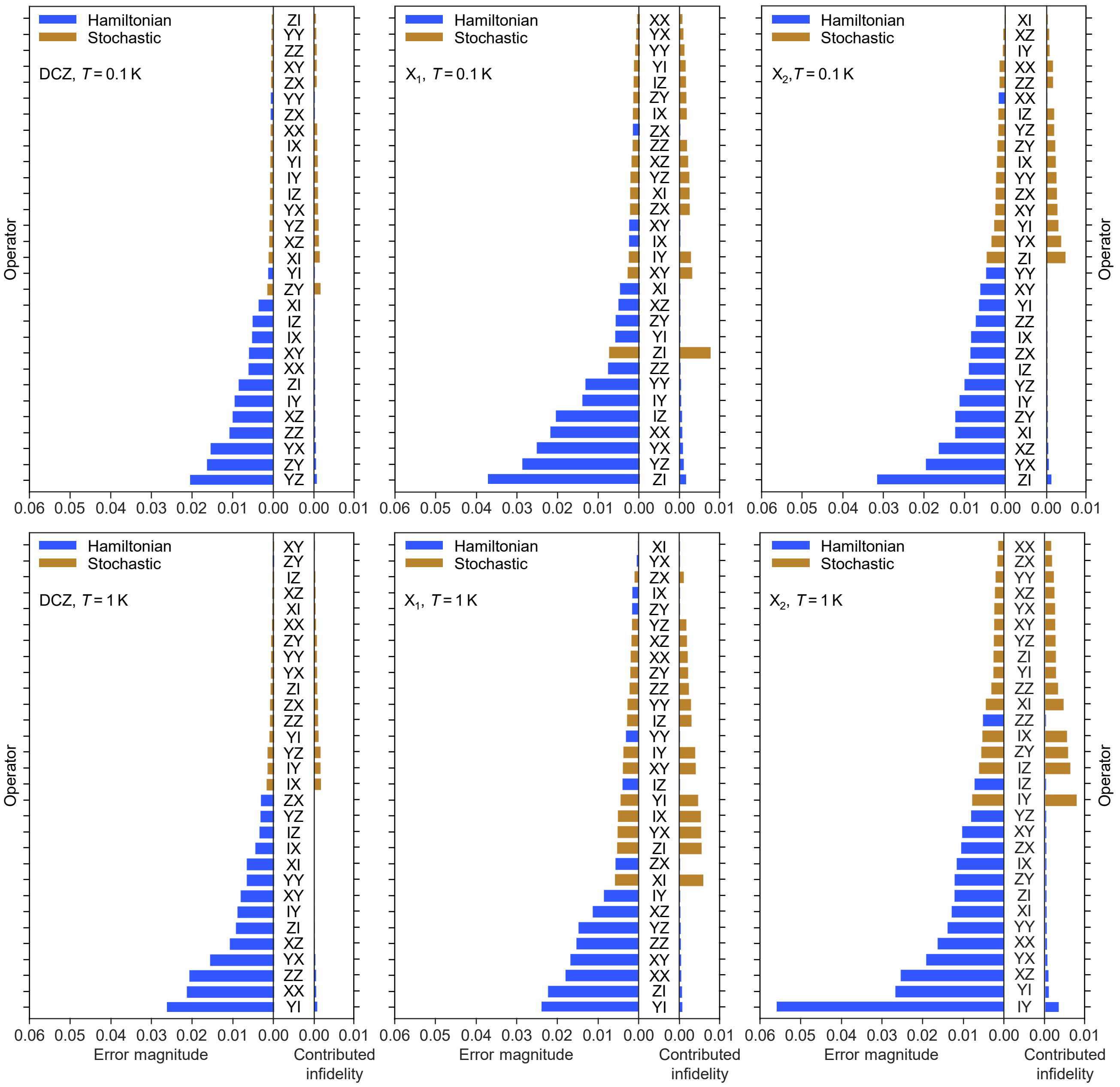

Supplementary Fig. 7 | All error channels obtained from the error generators. The bar charts detail the magnitudes of both Hamiltonian and stochastic errors, as well as their contribution to entanglement infidelities for the DCZ, $X_1(\pi/2)$, and $X_2(\pi/2)$ gates, at $B_0 = 0.79\,\mathrm{T}$, $T = 0.1\,\mathrm{K}$ and $1\,\mathrm{K}$. We identify that there is not a single stochastic error source that dominates the DCZ gate infidelities, unlike the single qubit gates. This means that it will be non-trivial to reduce the stochastic errors significantly. We attribute some of these errors to the slight differences in coupling strength each time a DCZ is executed. It would require advanced pulse engineering techniques [5–8] to eliminate the non-Markovian noise sources causing these inconsistencies. We also perceive an asymmetry in the error types: the errors on Q1 being very different from those on Q2. Despite the symmetric operation point [9–12] in the $(3, 3)$ charge state, the error symmetry may be convoluted by the local environmental factors of individual qubits, such as Si/SiO_2 interface roughness and charge noise [1]. If we could controllably choose the asymmetric operation in a particular dot direction, it would be potentially beneficial to QEC with tailored surface codes.

[1] Cifuentes, J. D. et al. Bounds to electron spin qubit variability for scalable CMOS architectures. Preprint at `https://doi.org/10.48550/arXiv.2303.14864`

[2] Yang, C. H. et al. Spin-valley lifetimes in a silicon quantum dot with tunable valley splitting. *Nat. Commun.* **4**, 2069 (2013).

[3] Leon, R. C. C. et al. Coherent spin control of s-, p-, d- and f-electrons in a silicon quantum dot. *Nat. Commun.* **11**, 797 (2020).

[4] Gilbert, W. et al. On-demand electrical control of spin qubits. *Nat. Nanotechnol.* **18**, 131–136 (2023).

[5] Yang, C. H. et al. Silicon qubit fidelities approaching incoherent noise limits via pulse engineering. *Nat. Electron.* **2**, 151–158 (2019).

[6] Khodjasteh, K. & Viola, L. Dynamically error-corrected gates for universal quantum computation. *Phys. Rev. Lett.* **102**, 080501 (2009).

[7] Kanaar, D. W., Güngördü, U. & Kestner, J. P. Two-qubit controlled-Z gates robust against charge noise in silicon while compensating for crosstalk using neural network. *Phys. Rev. B* **105**, 245308 (2022).

[8] Rimbach-Russ, M., Philips, S. G. J., Xue X. & Vandersypen, L. M. K. Simple framework for systematic high-fidelity gate operations. *Quantum Sci. Technol.* **8**, 045025 (2023).

[9] Xue X. et al. Quantum logic with spin qubits crossing the surface code threshold. *Nature* **609**, 343–347 (2022).

[10] Noiri, A. et al. Fast universal quantum gate above the fault-tolerance threshold in silicon. *Nature* **601**, 338–342 (2022).

[11] Reed, M. D. et al. Reduced sensitivity to charge noise in semiconductor spin qubits via symmetric operation. *Phys. Rev. Lett.* **116**, 110402 (2016).

[12] Martins, F. et al. Noise suppression using symmetric exchange gates in spin qubits. *Phys. Rev. Lett.* **116**, 116801 (2016).
